# Effect of Loading Changes on the Intraventricular Pressure Measured by Color M-Mode Echocardiography in Rats

**DOI:** 10.3390/diagnostics11081403

**Published:** 2021-08-03

**Authors:** Akira Yairo, Ahmed S. Mandour, Katsuhiro Matsuura, Tomohiko Yoshida, Danfu Ma, Pitipat Kitpipatkun, Konosuke Kato, Chieh-Jen Cheng, Hussein M. El-Husseiny, Takashi Tanaka, Kazumi Shimada, Lina Hamabe, Akiko Uemura, Ken Takahashi, Ryou Tanaka

**Affiliations:** 1 Laboratory of Veterinary Surgery, Department of Veterinary Medicine, Tokyo University of Agriculture and Technology, Tokyo 183-0054, Japan; akirayairo@gmail.com (A.Y.); k.matsuura.vet@gmail.com (K.M.); tomohiko7731-yoshida@yahoo.co.jp (T.Y.); dandanma1000@gmail.com (D.M.); pitipat_ki@rmutto.ac.th (P.K.); s154816x@st.go.tuat.ac.jp (K.K.); john_199328@yahoo.com.tw (C.-J.C.); hussien.alhussieny@fvtm.bu.edu.eg (H.M.E.-H.); bamse.rizea.vicky.polta@gmail.com (T.T.); ruiyue1221@gmail.com (K.S.); linahamabe@googlemail.com (L.H.); 2 Department of Animal Medicine (Internal Medicine), Faculty of Veterinary Medicine, Suez Canal University, Ismailia 41522, Egypt; 3 Department of Surgery, Anesthesiology and Radiology, Faculty of Veterinary Medicine, Benha University, Moshtohor, Toukh, Elqaliobiya 13736, Egypt; 4 Department of Veterinary Surgery, Division of Veterinary Research, Obihiro University of Agriculture and Veterinary Medicine, Obihiro 080-8555, Hokkaido, Japan; anco@vet.ne.jp; 5 Department of Pediatrics and Adolescent Medicine, Juntendo University Graduate School of Medicine, Tokyo 113-8421, Japan; kentaka@juntendo.ac.jp

**Keywords:** heart failure, color m-mode echocardiography, diastolic function, preload, intraventricular pressure

## Abstract

Evaluation of diastolic function is a pivotal challenge due to limitations of the conventional echocardiography, especially when the heart rate is rapid as in rats. Currently, by using color M-mode echocardiography (CMME), intraventricular pressure difference (IVPD) and intraventricular pressure gradient (IVPG) in early diastole can be generated and are available as echocardiographic indices. These indices are expected to be useful for the early diagnosis of heart failure (HF), especially diastolic dysfunction. There have not been any studies demonstrating changes in IVPD and IVPG in response to changes in loading conditions in rats. Therefore, the present study aims to evaluate CMME-derived IVPD and IVPG changes in rats under various loading conditions. Twenty rats were included, divided into two groups for two different experiments, and underwent jugular vein catheterization under inhalational anesthetics. Conventional echocardiography, CMME, and 2D speckle tracking echocardiography were measured at the baseline (BL), after intravenous infusion of milrinone (MIL, *n* = 10), and after the infusion of hydroxyethyl starch (HES, *n* = 10). Left ventricular IVPD and IVPG were calculated from color M-mode images and categorized into total, basal, mid-to-apical, mid, and apical parts, and the percentage of the corresponding part was calculated. In comparison to the BL, the ejection fraction, mid-to-apical IVPG, mid IVPG, and apical IVPD were significantly increased after MIL administration (*p* < 0.05); meanwhile, the end-diastolic volume, E-wave velocity, total IVPD, and basal IVPD were significantly increased with the administration of HES (*p* < 0.05). The increase in mid-to-apical IVPD, mid IVPD, and apical IVPD indicated increased relaxation. A significant increase in basal IVPD reflected volume overloading by HES. CMME-derived IVPD and IVPG are useful tools for the evaluation of various loading conditions in rats. The approach used in this study provides a model for continuous data acquisition in chronic cardiac disease models without drug testing.

## 1. Introduction

Heart failure (HF) is a major health problem with a prevalence of more than 23 million people worldwide caused by both diastolic and systolic dysfunctions [1,2]. Echocardiography is a simple and noninvasive method that allows the diagnosis of cardiovascular dysfunction in both animals and humans. Generally, the diastolic function contains two phases, namely myocardial relaxation and atrial contraction that create very complex hemodynamic changes in a short time. The conventional echocardiographic method is unable to detect diastolic insufficiency before the appearance of overt clinical symptoms, especially in animals with rapid heart rates such as rats [3,4]. Therefore, the accurate measurement of the left ventricle (LV) diastolic function mainly depends on intracardiac catheterization to evaluate stiffness constant *β*, *Tau*, and LV end-diastolic pressure.

With the rapid development of echocardiographic technology and the establishment of color M-mode echocardiography (CMME), the intraventricular pressure difference (IVPD) has begun to be used as a noninvasive index for cardiac function evaluation. IVPD is the pressure difference between different points within the LV generated in early diastole when the apical pressure becomes lower than the basal one [5]. The IVPD was invented by considering the blood flow dynamics inside the ventricle as measured by intracardiac catheterization. The IVPD can be estimated from CMME by calculating Euler equations; therefore, the sucking force in the ventricle can be determined noninvasively [6]. This allows calculation of the IVPD between any two points in the ventricle; hence, the LV can be divided into several segments for detailed evaluation. Furthermore, to exclude the effect of the LV length on IVPD, the IVPD is divided mathematically by the LV length to calculate the intraventricular pressure gradient (IVPG) [7]. IVPD obtained by CMME is considered a sensitive indicator of diastolic function [6] and has the advantage of repeatability, unlike invasive catheterization. Similarly, the IVPG exerts the same properties as IVPD without being affected by the LV length [8,9,10,11]. Generally, the length of the LV, from the mitral valve to the apex, is divided into basal, mid, and apical parts, and each part has its corresponding IVPD and IVPG indices and a defined role in the diagnosis of cardiac dysfunction. For instance, it was reported that basal IVPD increases when congestion progresses, whereas mid IVPD decreases when diastolic dysfunction progresses [8,12]. Mid-to-apical IVPG was found to reflect the active relaxation of the LV during diastole, whereas the apical IVPG plays the main role in actively sucking the blood into the ventricle [13,14].

Recently, many trials have been undertaken to incorporate these novel indices into cardiology research, particularly in diastology. Results revealed that *Tau*, the LV relaxation time constant measured by cardiac catheterization, was negatively correlated with total, basal, and mid IVPD under various loading states in dogs [8], while mid-to-apical IVPG was considered as a contractility index since it was well correlated with Emax, the contractility index, in chemotherapy-induced cardiomyopathy in dogs [9]. Total and mid-to-apical IVPG were identified as reliable predictors for children’s survival after chemotherapy [13]. Besides, in rat models, IVPG was assessed during the development and after medication of LV hypertrophy and uremic cardiomyopathy [10,15]. This innovation is expected to minimize the experimental time and limit the number of sacrificed animals due to invasive approaches. At the same time, when greater detection power of statistical analysis is necessary, more animals could be easily included because of the noninvasive nature of the technique. 

Rats play a central role as laboratory animal models for cardiovascular research including both diseases and drug testing since rats show good repeatability in the serial evaluation of cardiac function. Different echocardiographic approaches including parameters of diastolic function such as mitral inflow and tissue Doppler imaging could be implemented as in other animal models with larger heart sizes [16,17,18,19]. Recently, great attention has been directed toward the evaluation of diastolic function to understand its imperative role in the pathogenesis of HF for the early detection of cardiac events [20,21,22]. Although it can estimate volumes and blood and tissue velocities, the accuracy of diastolic function determined using the conventional echocardiographic technique is controversial since it can only indirectly reflects the intraventricular pressure or pressure–volume relationships. In addition, there is no single noninvasive index that provides a direct measurement of diastolic function including relaxation, restoring forces, compliance, or LV filling pressure, and a combination of different techniques is required [23,24]. Novel parameters such as IVPG and IVPD are worthy of consideration under various hemodynamic conditions. CMME-derived IVPD was evaluated in rats with diastolic dysfunction induced by diabetes mellitus [25,26]. Data from previous studies indicated that IVPD and IVPG are useful as noninvasive methods for repeated and continuous evaluation of the heart. The objective of the present study was to examine the usability of IVPD and IVPG in reflecting the cardiac loading conditions in rats after milrinone administration to increase cardiac contractility and hydroxyethyl starch infusion to increase the preload. 

## 2. Materials and Methods

### 2.1. Animal Preparation and Experimental Protocol

Twenty healthy 5- to 6-month-old male rats (Kitayama Labes, Nagano, Japan) weighing 530–777 g (average 654 g) were used in the present study and equally divided into two groups (*n* = 10 for each) for two different experiments. Animals were kept in an air-conditioned room at 25 ± 0.5 °C under a 12 h light/dark cycle, with the food and water provided ad libitum. After anesthetization of the rats with isoflurane, the thoraxes were shaved positioned in right and left lateral recumbency. Anesthesia was maintained by mask inhalation of isoflurane (concentration of 2.5%). A 24G intravenous catheter was introduced through the jugular vein. In the first experiment, after the acquisition of echocardiographic images at baseline (BL), milrinone (MIL) (Maruishi Pharmaceutical, Osaka, Japan) was administered intravenously at a constant rate of 10 µL kg^−1^ min^−1^ according to the previously reported protocol [27]. Echocardiographic examinations were repeated 20 min after MIL infusion. In the second experiment, after BL echocardiography, hydroxyethyl starch (HES) solution (6% Salinhes fluid solution, Fresenius Kabi Japan, Tokyo, Japan) was administered at a contrast rate of 5% of the total blood volume kg^−1^ min^−1^, and the echocardiographic images were acquired when the volume of administered HES reached 60% of the total blood volume [28]. Total blood volume was estimated using the following equation [29,30]:

BV (mL) = BW (gm) × 0.06 + 0.77(1)

Skin incision, as well as experimental time, were minimized, and all rats were humanely euthanized shortly after the experiment was accomplished and data were acquired. 

### 2.2. Conventional Echocardiography

F75CV ultrasonography system equipped with a sector probe of 10 MHz, UST52129 (Hitachi Aloka Medical Ltd., Tokyo, Japan), and supported with the two-dimensional speckle tracking and color M-mode echocardiography was used for all echocardiographic examinations according to the American Society of Echocardiography guidelines [30]. A full conventional echocardiographic protocol was performed including LV function from the standard right parasternal short-axis view at the papillary muscle level using M-mode. From this view, the LV end-diastolic diameter (LVIDd), end-diastolic volume (EDV), end-systolic volume (ESV), ejection fraction (EF%), and fraction shortening (FS%) were obtained. In addition, a Doppler assessment of the aortic blood flows from the apical four-chamber view was also performed. The mitral inflow indices, including early (E) and late (A) velocities and E/A ratio, were obtained using pulsed-wave Doppler echocardiography from the left apical four-chamber view. From the same view, tissue Doppler imaging mode was used to trace the systolic (s′) and early diastole (e′) velocities at the lateral and septal annulus of the mitral leaflets. Cardiac output (CO) was calculated using Pombo methods. E/lateral e′ was calculated by dividing E-wave velocity by lateral e′, and E/septal e′ was calculated by dividing E-wave velocity by septal e′. Stroke volume (SV) was calculated from aortic blood flow and aortic diameter. 

### 2.3. Two-Dimensional Speckle Tracking Echocardiography (2DSTE)

The 2DSTE data were obtained from the standard apical four-chamber view. ECG was attached and the entire cardiac cycle was captured and recorded as a movie using UST52129 (Hitachi Aloka Medical Ltd., Tokyo, Japan). After acquiring the movies, 2DSTE analysis was performed using DAS-RS1 software (Hitachi Aloka Medical, Tokyo, Japan), and the global longitudinal strain (GLS) and strain rate (SR) were measured [31].

### 2.4. Color M-Mode Echocardiography (CMME)

The CMME was used for the evaluation of IVPD and IVPG. The ultrasound machine was set to sweep speed of 300 mm/s and color baseline-shift of −64 to increase the Nyquist limit for proper tracing of the CMME. Firstly, the left apical 4-chamber view was adjusted and the blood flow tract from the left atrium toward the LV apex across the mitral valve was optimized. After that, the M-mode was initiated to trace the inflow. Color M-mode images were saved for further offline analysis using MATLAB (The MathWorks, Natick, MA, USA). IVPD derived from CMME was previously validated against the temporal IVPD obtained by the catheterization method using a micromanometer in animal experiments [7]. For software analysis of location-dependent IVPD, the LV was divided into three parts: the basal part expressed as basal IVPD (BIVPD) that matched the pressure at the mitral valve, the apical part known as apical IVPD (AIVPD) at the apex of the LV, and the remaining central part named mid IVPD (MIVPD). The total pressure was defined as total IVPD (TIVPD) and the percentage of each contributing part of IVPD in relation to the total IVPD was calculated. LV length was obtained from the left parasternal long-axis view. The intraventricular pressure gradient (IVPG) was calculated as follows [10,25]:

(2) $$\mathrm{IVPG}\;(\mathrm{mmHg}/\mathrm{cm})=\frac{\mathrm{IVPD}}{\mathrm{LV}\;\mathrm{length}}$$

### 2.5. Statistical Analysis

Wilcoxon matched-pairs signed-rank test was used to compare parameters of baseline (BL) and after intravenous administration of milrinone (MIL) or hydroxyethyl starch (HES) using GraphPad Prism Version 8 (GraphPad Software Inc., San Diego, CA, USA). Results are expressed as median with data range. *p* < 0.05 value was considered statistically significant.

## 3. Results

### 3.1. Conventional Echocardiography

The effect of MIL and HES on heart functions measured by conventional echocardiography is shown in Table 1. MIL infusion, compared with the BL, resulted in significant decreases in LVIDd (8.41 vs. 7.75 mm, *p* < 0.05), EDV (0.53 vs. 0.47 mL, *p* < 0.05), LVOT (114 vs. 99.8 cm/s, *p* < 0.05), SV (0.8 vs. 0.72 mL, *p* < 0.05), and LV length (1.12 vs. 1.03 cm, *p* < 0.001) and no significant change in ESV, E velocity, and septal and lateral E/e′. On the other hand, HES significantly increased LVIDd (6.97 vs. 7.66 mm, *p* < 0.05), EDV (0.43 vs. 0.65 mL, *p* < 0.01), SV (0.62 vs. 0.86 mL, *p* < 0.01), E-wave (74.6 vs. 122 cm/s, *p* < 0.01), and E/e′ of the septal (14.8 vs. 21.1, *p* < 0.01) and lateral (13.4 vs. 16.1, *p* < 0.01) annuli compared with the BL. Moreover, both MIL and HES significantly increased EF percentage and FS percentage (*p* < 0.05) but did not significantly alter the HR, septal s′, and lateral s′. 

### 3.2. Two-Dimensional Speckle Tracking Echocardiography

**Figure 1:**
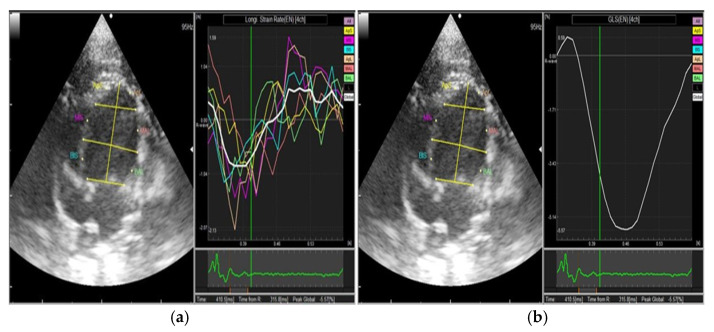
Two-dimensional speckle tracking echocardiography (2DSTE) in rats obtained from the left apical 4-chamber view. The entire left ventricular endocardium was traced for evaluation of strain rate (SR, **a**) and global longitudinal strain (GLS, **b**).

**Figure 2:**
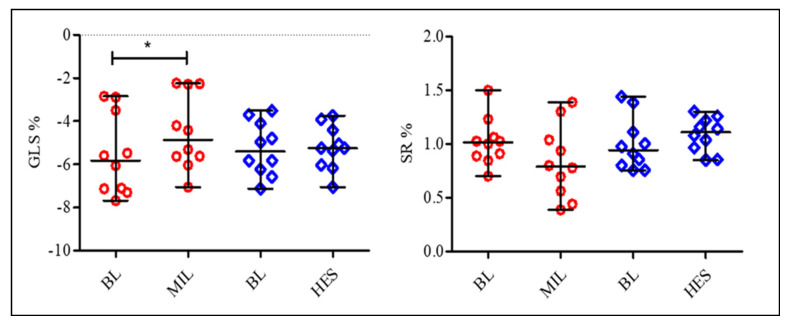
Effect of various loading conditions on 2DSTE measurements. Plots showing the median (central horizontal line) and range (*n* = 10, for each experiment). BL, baseline; MIL, milrinone; HES, hydroxyethyl starch; SR, strain rate; GLS, global longitudinal strain. * *p* < 0.05 compared with BL.

The results of the 2DSTE are presented in Figure 1 and Figure 2. GLS was significantly increased after MIL administration (−5.82% vs. −4.87%, *p* < 0.05); however, SR and GLS were not significantly increased after HES administration compared with the BL.

### 3.3. Color M-Mode Echocardiography

#### 3.3.1. Effect of Loading Condition on IVPD

**Figure 3:**
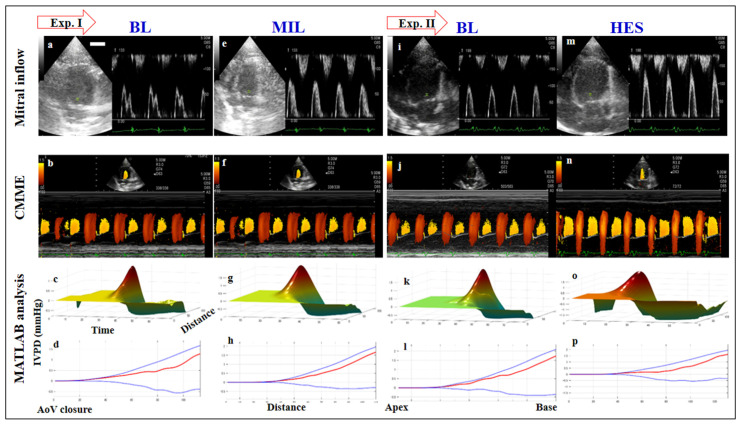
The echocardiography and IVPD (IVPG) in rats. Images in each column were captured from the same rat. In the first experiment, the heart function was evaluated at the baseline (**a**–**d**) and after milrinone (MIL, **e**–**g**). In the second experiment, measurements were recorded at the baseline (BL, **i**–**l**) and after hydroxyethyl starch infusions (HES, **m**–**p**). Firstly, the mitral inflow was evaluated in all groups by pulsed-wave Doppler echocardiography from the left apical 4-chamber view (**a**,**e**,**i**,**m**) that showed significantly increased E-wave after HES infusion. Color m-mode echocardiography (CMME) was switched on and photos were captured (**b**,**f**,**j**,**n**) for further calculation. The IVPD was calculated by MATLAB software using in-house coding, and the three-dimensional temporal and spatial profiles of the left ventricular IVPD was obtained where the time, IVPD, and distance were determined (**c**,**g**,**k**,**o**). Subsequently, the IVPD (IVPG) in early diastole was identified (**d**,**h**,**i**,**p**). The top (blue), middle (red), and bottom (blue) lines represent inertial, total, and convective IVPD, respectively.

The data of IVPD are presented in Table 2 and Figure 3. After MIL administration, there was a significant increase in mid-to-apical IVPD (0.71 vs. 0.79 mmHg, *p* < 0.01), MIVPD (0.58 vs. 0.66 mmHg, *p* < 0.01), AIVPG (0.14 vs. 0.16 mmHg, *p* < 0.05), and the percentages of mid-to-apical IVPD (35.9 vs. 41.1%, *p* < 0.05) and MIVPD (28.7 vs. 34.8%, *p* < 0.01) compared with the baseline. Meanwhile, TIVPD showed no change after MIL infusion. Concerning HES infusion, there was a significant increase in TIVPD (1.6 vs. 2.34 mmHg, *p* < 0.01), BIVPD (0.99 vs. 1.59 mmHg, *p* < 0.01), AIVPD (0.1 vs. 0.16 mmHg, *p* < 0.5), and the percentage of BIVPD (61.9 vs. 69.8, *p* < 0.010) compared with the BL. Mid-to-apical IVPD and mid IVPD were increased after HES infusion compared with the BL but not statistically significantly. The percentages of mid-to-apical IVPD and MIVPG were significantly reduced after HES infusion.

#### 3.3.2. Effect of Loading Condition on IVPG

The data of IVPG are shown in Table 2 and Figure 3. In the same way as IVPD results, in response to MIL administration, there was a significant increase in mid-to-apical IVPG (0.64 vs. 0.81 mmHg, *p* < 0.01), MIVPG (0.51 vs. 0.66 mmHg, *p* < 0.01), AIVPG (0.13 vs. 0.16 mmHg, *p* < 0.05), and percentage of AIVPG (7.25 vs. 8.46 mmHg, *p* < 0.05) compared with the baseline. The TIVPG and BIVPG showed no change after MIL infusion. Regarding the HES administration, there was a significant increase in TIVPG (1.53 vs. 1.98 mmHg) and BIVPG (0.92 vs. 1.44 mmHg) in comparison to the BL (*p* < 0.01); meanwhile, mid-to-apical IVPG, MIVPG, AIVPG, and percentage of AIVPG showed no statistical difference.

## 4. Discussion

Rats are useful models for cardiovascular research because various echocardiographic techniques can be performed. Preparation of chronic heart failure models and their sequential evaluation is essential to establish various treatment protocols to improve diastolic dysfunction such as heart failure with preserved ejection fraction (HFpEF). The time constant τ, which was derived from a pressure–volume loop, has been recognized as the gold standard of evaluation of diastolic function. However, this invasive method is not suitable for repetitive measurement, and most studies using rat models of heart failure were limited by a comparative study in the short term and did not study the long-term prognosis, in addition to the high cost of the needed catheter [32,33]. In contrast, echocardiography is suitable for repetitive measurement because it is minimally invasive; nevertheless, conventional parameters have a shortcoming in detecting diastolic dysfunction, and a novel parameter needs to be established [8,24]. This requires the incorporation of novel diagnostic tools used for diastolic function evaluation. Recently, CMME-derived IVPD and IVPG showed breakthroughs in the diagnosis of diastolic dysfunction and are suggested to be included in the conventional echocardiography protocol [9]. In the present study, we studied the response of IVPD and IVPG indices to pharmacologically induced alteration in the loading conditions in rats.

Several echocardiographic parameters have been reported to reflect diastolic function. For example, early diastolic mitral annular velocity (e′) and maximal flow velocity during early diastole divided by maximal flow atrial systole (E/A) are frequently used clinically in humans and animals. However, E/A is not available when E-wave velocity and A-wave velocity of the mitral inflow are fused. EA fusion has been observed in 50% of rats in the current experiment; therefore, the E/A ratio was not reported since it cannot reflect the diastolic function evaluation in the investigated groups. EA fusion is common in rats as shown previously in other studies at approximately similar heart rates [10,26]. When the HR is increased, diastasis is disturbed, leading to transmitral inflow pattern change to EA fusion pattern or half separation instead of E–A separation. Basically, the E-wave velocity is dependent on LA–LV pressure gradient (LV relaxation and LA pressure) in early diastole; meanwhile, the A-wave velocity depends on LA–LV pressure gradient in late diastole (LV stiffness and LA contractility). However, the increase in the E/A ratio is useful in patients with myocardial disease since it can predict the increase in LV filling pressure but is not useful in normal subjects [23]. Because of these limitations, in the present study, IVPD and IVPG were featured in a verification experiment for the evaluation of diastolic function under different loading conditions. IVPD is a parameter that reflects the sucking force in the LV, characterized by rapid deformation of the LV wall followed by elastic recoil, which is discharged as elastic energy accumulated by contraction of the LV during end-systole [34]. Therefore, IVPD is expected to have a certain relationship with the contractile and/or relaxation reserve and will be affected by loading conditions.

Milrinone is well known as a positive inotrope and vasodilator with little chronotropic activity. It also provides a significant lusitropic effect on the heart, which is extremely important for the heart with single ventricle pathophysiology [35]. These properties of MIL explain the increase in EF and FS [36], but the return flow to the heart does not match the increased cardiac positive inotropic change; hence, SV, CO, and LVOT were reduced after MIL infusion. Intravenous administration of milrinone at low doses has a pulmonary vasodilator effect through a reduction in pulmonary vascular resistance and increases the pulmonary blood flow, which reduces the venous return and diastolic parameters [37].

Some deviation of the obtained echocardiographic parameters was observed, which may be related to the change in HR, which can be easily changed according to the change in preload or sympathetic excitement. Each group contains a variety of hemodynamics; hence, some rats showed increased contraction with adequate preload, and others showed increased contraction with less preload but no deviation in the individual rat. Our results also showed a significant increase in apex-related IVPG indices (MIVPG, mid-to-apical IVPG, and AIVPG) after MIL administration. These parameters reflect the increase in sucking force and diastolic function of the LV [36,38]. However, TIVPG did not reflect the increase in apex-related IVPG. In the same way, BIVPG, which was not significantly reduced, would explain the discordance between TIVPG and diastolic function observed in this study. In our previous study, the decrease in BIVPG in dogs was induced by reduced congestion attributed to a positive inotropic effect of MIL [39]. Only IVPG was able to detect a hemodynamic change during MIL infusion in a comprehensive way because when IVPG is generated, it reflects all the myocardial changes, such as torsion, untwisting rate, and a change in longitudinal strain and circumference strain, which are crucial in diastolic function evaluation [14,23]. On the other hand, e′, which showed no change in the current experiment after MIL and HES infusion, can capture only a maximum velocity of one-dimensional annular motion, and 2DSTE, which is considered a useful tool for early diagnosis of cardiac dysfunction [40], has limitation with time resolution under a high heart rate [41]. Intracardiac blood flow dynamics is a product generated from cardiac wall motion [42], and the secret of the high sensitivity of IVPG derives from the measurement of intracardiac blood flow with high time resolution using color M-mode [8,43,44].

Administration of HES will increase venous return and cardiac output [28]. EF is greatly increased after HES infusion. This may be related in part to the observed increase in EDV resulting in the increased calculated EF. Besides, based on the Frank–Starling law, as a larger volume of blood flows into the ventricle, the blood stretches the cardiac muscle fibers, leading to an increase in the force of contraction. The significantly increased EDV, E-wave velocity, and septum E′/e′ indicated an increased preload. E/e′ is a parameter of left atrial pressure and can be measured at the two points of the mitral annulus, septal and lateral [45]. Under a preloading effect, in response to HES administration, both septum and lateral E’/e’ were significantly increased, but they did not reflect the preloading effect with MIL. Variations in acute preloading effect on septal e′ and lateral e′ waves were previously reported [41]; therefore, based on our observation of the current study, evaluation of left atrial pressure using E′/e′ has some limitations since these waves were not solid in response to HES and MIL infusion. Consequently, BIVPG is more consistent and suitable for monitoring the preload after MIL administration.

The advantage of IVPD is that it shows the pressure gradient at any segment. In the present study, the heart was evenly divided into three parts for evaluation [10,25]; however, it is possible to divide the LV into any number of parts as necessary. In this experiment, the basal IVPD reflected congestion, and the apical part of IVPG reflected a change in diastolic function. Unlike the E-wave, which is greatly influenced by both congestion and diastolic function, IVPG is available to evaluate congestion and diastolic function separately by a single measurement of IVPG. After HES administration, TIVPG, which was not significantly different after MIL administration, was significantly increased. This result was in agreement with a previous report in which the pulmonary capillary wedge pressure and the IVPG were positively correlated during exercise, suggesting that IVPG was affected by left atrial pressure [11]. In other words, TIVPD is affected by left atrial pressure, so TIVPD is not suitable for the assessment of diastolic function. 

Change in the LV length was reported in the current study under MIL and HES administration. A previous study showed that LV length was positively correlated with IVPD but not associated with IVPG [11]. IVPD was also reported to be sensitive to heart length within a single species [8,9]. As indicated by these reports, it is reasonable to use IVPG in experimental systems to exclude the effect of LV length, especially when the LV length changes due to medication. However, in the present study, both IVPD and IVPG showed similar results, and in some segments, only IVPD showed significant differences. It is difficult to conclude clearly that IVPG is more useful than IVPD. IVPD has an advantage in that it shows actual pressure difference, which offers a physiological indicator for the evaluation of cardiac pathophysiology, whereas IVPG shows the pressure slope in the ventricle.

We also measured the percentage of IVPD, the ratio of each segment related to the total IVPD. By eliminating the effect of individual differences in body size, the percentage of IVPG was expected to have some advantages. In the current study, the percentage of BIVPD was decreased after MIL administration and increased after HES administration. Additionally, the percentage of mid-to-apical IVPD and the percentage of MIVPD showed opposite reactions after MIL administration and HES administration. These results revealed that these indices are also useful for the evaluation of cardiac status after drug administration. 

Some limitations of the present study should be considered. We did not perform intracardiac catheterization for hemodynamic assessment. The number of used rats was low (*n* = 10) for each experiment; however, the derived data were sufficient to support our hypothesis. Besides, we did not examine the heart function at the baseline with the vehicle of the HES. We also did not evaluate the body temperature during the examination to exclude the effect of temperature on our measurements; nevertheless, we ensured minimal operation time and adequate warming of rats during the experiment to reduce environment-induced temperature and hemodynamic alterations.

## 5. Conclusions

For animals with rapid heart rates such as rats, noninvasive parameters that reliably reflect diastolic function, such as IVPD and IVPG, are valuable. IVPD and IVPG are more sensitive to the changes caused by the administration of MIL and HES, and their availability compared with conventional echocardiography parameters and STE was demonstrated in this study. The results showed that IVPD and IVPG are useful as noninvasive indices in the short-term evaluation and are expected to be validated for long-term study of diastolic function and congestion. IVPD and IVPG are expected to contribute to experiments for continuous data acquisition in chronic heart disease models without drug testing. 

## Figures and Tables

**Table 1:** Effect of various loading conditions on conventional echocardiographic parameters.

Variables	Experiment I		Experiment II	
	BL	MIL	BL	HES
LVIDd (mm)	8.41 (7.10–10.6)	7.75 (6.20–9.37) *	6.97 (6.9–9.40)	7.66 (7.30–10.1) ^†^
EDV (mL)	0.53 (0.37–1.20)	0.47 (0.23–0.83) *	0.43 (0.33–0.83)	0.65 (0.60–1.03) ^††^
ESV (mL)	0.10 (0.1–0.27)	0.10 (0.1–0.10)	0.13 (0.1–0.37)	0.10 (0.08–0.20)
CO (L/min)	0.07 (0.03–0.13)	0.05 (0.03–0.32)	0.07 (0.04–0.17)	0.08 (0.04–0.17)
EF (%)	78.7 (57.4–84.3)	84.9 (72.1–89.2) *	72.8 (57.8–87.3)	87.7 (74.8–93.3) ^†^
FS (%)	40.3 (25.2–46.6)	47.1 (34.7–52.4) **	35.2 (24.8–50.0)	44.2 (36.8–60.0) ^†^
E (cm/s)	80.7 (66.3–102)	76.3 (71.3–85.5)	74.6 (65.7–104)	122 (83.1–138^) ††^
Septal s′ (cm/s)	4.65 (3.88–5.03)	4.45 (3.93–5.03)	5.08 (3.30–5.73)	4.53 (3.93–5.15)
Septal e′ (cm/s)	4.95 (4.53–6.43)	5.03 (4.23–7.93)	4.98 (3.97–9.40)	5.47 (4.70–6.57)
E/Septal e′	17.0 (14.1–21.0)	15.5 (9.54–20.5)	14.8 (10.8–19.2)	21.1 (16.1–26.0^) ††^
Lateral s′ (cm/s)	5.32 (3.87–6.63)	5.68 (3.87–6.77)	5.85 (4.47–6.37)	5.58 (5.37–7.03)
Lateral e′ (cm/s)	5.60 (3.77–7.57)	6.15 (3.60–7.83)	5.70 (4.57–9.47)	7.43 (5.90–8.40)
E/Lateral e′	15.9 (10.8–19.8)	16.0 (10.6–18.7)	13.4 (9.95–14.5)	16.1 (12.9–18.8) ^††^
LVOT (cm/s)	114 (78.7–136)	99.8 (85.9–121) *	90.0 (73.2–161)	116 (83.9–144)
SV (mL)	0.80 (0.50–0.90)	0.72 (0.60–0.80) *	0.62 (0.37–1.07)	0.86 (0.60–1.07) ^††^
HR (bpm)	307 (228–457)	387 (259–466)	247 (234–388)	280 (221–325)
LV (cm)	1.12 (0.93–1.45)	1.03 (0.91–1.38) **	0.89 (0.80–1.37)	1.04 (0.64–1.37)

Values are presented using the median with data range. Comparison was done between baseline and after medication within the same group (*n* = 10, for each experiment). * used to compare between baseline (BL) and milrinone (MIL; * *p* < 0.05, ** *p* < 0.01). ^†^ fitted to compare between BL and hydroxyethyl starch (HES; ^†^ *p* < 0.05, ^††^ *p* < 0.01). LVIDd, left ventricular internal diameter at diastole; EDV, end-diastolic volume; ESV, end-systolic volume; CO, cardiac output; EF, ejection fraction; FS, fractional shortening; E, peak velocity of early diastole; Septal s′ and e′, tissue Doppler of systolic and early diastolic wave velocities at septal mitral annulus; Lateral s′ and e′, tissue Doppler of systolic and early diastolic wave velocities at the lateral mitral annulus; LVOT, LV outflow tract; SV, stroke volume; HR, under anesthesia heart rate; LV, left ventricular length measured from closed mitral valve (early systole) to apex in apical four-chamber view.

**Table 2:** Changes in IVPD and IVPG under various loading conditions**.**

Variables	Experiment I		Experiment II	
	BL	MIL	BL	HES
TIVPD (mmHg)	2.13 (1.19–4.31)	2.01 (1.35–4.30)	1.60 (1.10–2.55)	2.34 (1.89–2.84) ^††^
BIVPD (mmHg)	1.36 (0.68–2.42)	1.34 (0.71–2.30) *	0.99 (0.55–1.78)	1.59 (1.21–2.06) ^††^
Mid-to-apical IVPD (mmHg)	0.71 (0.51–1.89)	0.79 (0.61–1.97) **	0.64 (0.44–0.78)	0.75 (0.47–0.88)
MIVPD (mmHg)	0.58 (0.41–1.50)	0.66 (0.51–1.52) **	0.54 (0.34–0.66)	0.59 (0.36–0.71)
AIVPD (mmHg)	0.14 (0.08–0.39)	0.16 (0.09–0.44) *	0.10 (0.05–0.14)	0.16 (0.05–0.21) ^†^
% of BIVPD	64.1 (56.2–74.1)	58.9 (52.5–62.2) **	61.9 (49.6–69.5)	69.8 (58.1–81.4) ^††^
% of mid-to-apical IVPD	35.9 (31.3–43.8)	41.1 (37.8–47.5) **	38.1 (30.5–50.5)	30.2 (18.6–41.9) ^††^
% of MIVPD	28.7 (20.8–34.8)	34.8 (29.0–38.7) **	32.2 (25.9–41.8)	22.7 (14.1–33.9) ^††^
TIVPG (mmHg)	1.89 (1.28–2.97)	1.996 (1.42–3.09)	1.53 (1.35–2.08)	1.98 (1.70–2.59) ^††^
BIVPG (mmHg)	1.21 (0.74–1.67)	1.21 (0.75–1.66)	0.92 (0.68–1.44)	1.44 (1.09–1.74) ^††^
Mid-to-apical IVPG (mmHg)	0.64 (0.51–1.30)	0.81 (0.60–1.42) **	0.62 (0.52–0.72)	0.64 (0.36–0.85)
MIVPG (mmHg)	0.51 (0.41–1.03)	0.66 (0.51–1.10) **	0.52 (0.44–0.59)	0.52 (0.28–0.73)
AIVPG (mmHg)	0.13 (0.08–0.27)	0.16 (0.09–0.32) *	0.09 (0.08–0.13)	0.14 (0.05–0.16)
% of AIVPG	7.25 (4.06–9.02)	8.46 (5.98–10.5) *	6.06 (4.62–8.70)	6.06 (2.64–9.61)

Color M-mode derived echocardiographic variables in rats during the increase in preload. Comparison was done between baseline and after medication within the same group. Values are presented using the median with data range (*n* = 10, for each experiment). * used to compare between baseline (BL) and milrinone (MIL; * *p* < 0.05, ** *p* < 0.01). † fitted to compare between BL and hydroxyethyl starch (HES; ^†^ *p* < 0.05, ^††^ *p* < 0.01). TIVPG, total intraventricular pressure gradient; BIVPG, basal intraventricular pressure gradient; mid-to-apical IVPG, middle-to-apical intraventricular pressure gradient; MIVPG, middle intraventricular pressure gradient; AIVPG, apical intraventricular pressure gradient; TIVPD, total intraventricular pressure difference; BIVPD, basal intraventricular pressure difference; Mid-to-apical IVPD, middle-to-apical intraventricular pressure difference; MIVPD, middle intraventricular pressure difference; AIVPD, apical intraventricular pressure difference; % of BIVPD, the percentage of basal intraventricular pressure difference to total intraventricular pressure difference; % of mid-to-apical IVPD, the percentage of middle-to-apical intraventricular pressure difference to total intraventricular pressure difference; % of MIVPG, the percentage of middle intraventricular pressure difference to total intraventricular pressure difference; % of AIVPG, the percentage of apical intraventricular pressure difference to total intraventricular pressure difference.

## Data Availability

The data presented in this study are available on request.
